# Diffusion tensor imaging at 3T for diagnosing root avulsion in adults with acute traumatic brachial plexus injuries

**DOI:** 10.1016/j.nicl.2025.103806

**Published:** 2025-05-21

**Authors:** Ryckie G. Wade, Irvin Teh, David Shelley, Robert D. Bains, James D. Bedford, Lucy E. Homer Newton, Chye Yew Ng, Grainne Bourke

**Affiliations:** a Leeds Institute for Medical Research, University of Leeds, Leeds, UK; b Department of Plastic, Reconstructive and Hand Surgery, Leeds Teaching Hospitals Trust, Leeds, UK; c Leeds Institute for Cardiovascular and Metabolic Medicine, University of Leeds, Leeds, UK; d The Advanced Imaging Centre, Leeds Teaching Hospitals Trust, Leeds, UK; e Department of Plastic Surgery & Burns, Manchester University NHS Foundation Trust, UK; f Wrightington Wigan and Leigh NHS Foundation Trust, Wigan, UK

**Keywords:** Diffusion, Magnetic resonance, DTI, Nerve injury, Brachial plexus

## Abstract

**Background:**

Root avulsion in patients with traumatic brachial plexus injury (tBPI) are common and MRI is used to help identify patients who need urgent reconstruction. Diffusion tensor MRI (DTI) generates proxy measures of nerve ‘health’ which are sensitive to myelination, axon diameter, fibre density and organisation. This prospective multicentre pilot study assessed the utility of DTI for detecting root avulsion in adults with acute traumatic brachial plexus injury.

**Methods:**

Patients underwent DTI at 3 Tesla. Fractional anisotropy (FA) and radial diffusivity (RD) were extracted from spinal nerve roots. The reference standard was surgical exploration or surveillance if spontaneous recovery occurred preoperatively. Comparisons were made between spinal nerve root avulsions, in-continuity roots and the contralateral uninjured roots, using linear methods and 95% confidence intervals (CI) were computed.

**Results:**

14 males with tBPI (mean age 44 years, SD 14) were scanned at a mean 18 days post-injury (CI 15–21). Diffusion was more isotropic in avulsed roots; root avulsions had 12 % lower FA than injured in-continuity roots (CI 5–19) and 14 % lower FA (CI 7–21) than the contralateral uninjured side. Similarly, avulsed roots had higher radial diffusivity than injured in-continuity roots (mean difference 0·30 x10^−3^ mm^2^/s [CI 0·01–0·60]) and contralateral uninjured roots (mean difference 0·36 x10^−3^ mm^2^/s [CI 0·7–0·64]).

**Conclusions:**

Diffusion tensor imaging appears to be sensitive to early microstructural changes in the distal stumps of avulsed roots in adults with tBPI. DTI may supplement morphological MRI to better identify patients who need early reconstruction.

## Highlights

• DTI is sensitive to early microstructural changes in traumatic brachial plexus injury root avulsions.
• Avulsed spinal nerve roots exhibit more isotropic diffusion days-to-weeks after injury.
• The time from injury to scanning did not change DTI parameter estimates.

## 1 Introduction

Traumatic brachial plexus injuries (tBPI) affect 1.2 % of patients involved in major trauma (Midha, 1997, Zaidman et al., 2024, Boyle et al., 2025). These life-changing injuries typically affect young adults and cause disability (Franzblau et al., 2014, Dolan et al., 2012), pain (Teixeira et al., 2015), psychological morbidity (Franzblau and Chung, 2015, Christy et al., 2024) and impaired quality of life (Franzblau et al., 2014, Dolan et al., 2012). Consequently, most cannot return to their original occupation (Brown et al., 2023) and suffer personal costs exceeding $1million over their lifetime, with 32 % experiencing catastrophic healthcare expenses (Kay et al., 2023).

The most common form of tBPI from major trauma is root avulsion (Wade et al., 2019). In this situation, restoration of limb function requires nerve transfer(s), which are low morbidity cost-effective procedures (Wali et al., 2017) that significantly improve function (Yang et al., 2012). Early diagnosis of root avulsion is critical because early reconstruction improves outcomes (Martin et al., 2019, Jivan et al., 2009). Each month of delay to reconstruction reduces the probability of useful motor function by 7 % (Lee et al., 2023). Also, early diagnosis and surgery might mitigate the chronic neuropathic pain (Makin et al., 2013) which is experienced by 95 % of patients (Teixeira et al., 2015). Therefore, early and accurate diagnosis of root avulsion is of paramount importance.

MRI is the best non-invasive test for tBPI in adults, although morphological imaging misclassifies approximately 28 % of in-continuity nerves as avulsed and fails to identify approximately 7 % of true avulsions (Wade et al., 2019). Supplementing standard morphological imaging with quantitative techniques could improve the overall accuracy. Diffusion-weighted MRI provides objective information about the microstructure of tissue and so the health of peripheral nerves. The diffusion tensor (diffusion tensor imaging, DTI) is the most common method of modelling the diffusion propagator and DTI metrics are sensitive to axon type, diameter, myelination, density and organisation (Heckel et al., 2015, Andersson et al., 2018, Friedrich et al., 2020), with the fascicle being the most anisotropic compartment (Pušnik et al., 2023). DTI has diagnostic utility in compression neuropathy (carpal tunnel syndrome (Rojoa et al., 2021), cubital tunnel syndrome (Griffiths et al., 2020, Gottfried et al., 2015, Ho et al., 2018, Park et al., 2020, Altun et al., 2013, Iba et al., 2010), lumbosacral root compression from disc herniation (Liang et al., 2021, Wang et al., 2022), inflammatory (Kronlage et al., 2017) and degenerative neuropathies such as Charcot-Marie-Tooth disease (Sun et al., 2022, Cheah et al., 2021, Kim et al., 2019) amongst others. DTI is also exquisitely sensitive to traumatic peripheral nerve injuries (Pridmore et al., 2021) because the tissues destruction, associated inflammation and haemorrhage are more profound (Pridmore et al., 2021) and detectable within days (Farinas et al., 2020).

To-date, several studies have reported DTI in the healthy adult brachial plexus (Oudeman et al., 2018, Ho et al., 2017, Vargas et al., 2010, Tagliafico et al., 2011, Gasparotti et al., 2013, Payen et al., 2023) such that there are now normative values available for a limited range of acquisition settings (Wade et al., 2020). Two studies have reported on the use of DTI in tBPI; however, this was limited to chronic injuries in one article (Wade et al., 2020) and in the other, only on the agreement between deterministic tractography and morphological imaging, without reporting DTI metrics (Gasparotti et al., 2013). The absence of studies examining the utility of DTI in acute tBPI underpins the rationale for this study.

## 2 Methods

This multicentre cohort study was designed and reported in accordance with the STROBE and STARD guidance, taking into account the domains of the QUADAS-2 (Whiting et al., 2003) and PRISMA-DTA (McGrath et al., 2017) tools. Approval was provided by the National Health Research Authority (ID 19/NW/0324) and written informed consent was obtained from participants.

### 2.1 Objectives

The primary objective was to determine whether DTI was sensitive to the early microstructural changes caused by traumatic avulsion of the roots of the brachial plexus. Secondarily, we planned to i) explore the relationship between the delay to scanning (time from injury to MRI) and DTI parameters, to understand if there is an ‘ideal time’ to scan post-injury, ii) assess the agreement between region-of-interest (RoI) and tract-derived DTI parameters to understand whether the extraction technique biases diffusion parameter estimates.

### 2.2 Recruitment

Between April 2019 and September 2021, 23 adults with tBPI were treated as in-patients within three centres (Leeds, Manchester and Wrightington) in the North of England. Of these, 3 were ineligible (major vascular injuries which warrant immediate reconstruction) and 7 declined (due to claustrophobia, pandemic related anxiety or without reason).

### 2.3 MRI acquisition

Imaging was performed at a field strength of 3T (3T) using a MAGNETOM Prisma (Siemens Healthcare, Erlangen, Germany). Participants were scanned supine using a 64-channel head & neck coil, and 18-channel body coil. DTI was acquired using single-shot echo-planar imaging with the following parameters: 50 contiguous 2.5 mm axial slices, in-plane resolution 2·5mm^2^, field-of-view (FoV) 305 × 305 mm (matrix size 122 × 122), a TrueForm B1 shim, 64 non-collinear monopolar diffusion-encoding gradients, b-value 1000  s/mm^2^, 4 interleaved non-DW (b0) images, TE 68 ms, TR 6500 ms, GRAPPA 2, 6/8 partial Fourier, receiver bandwidth 2275 Hz, distortion correction off and strong fat saturation. Four repetitions (256 diffusion-weighted images and 16 interleaved b0s) were acquired over 24 min. This was supplemented by T2w imaging for clinical reporting (breath-gated, fat and blood supressed, contiguous STIR SPACE, and myelography acquired by constructive-interference in steady state).

### 2.4 Preprocessing

DICOMs were converted to nifti using dcm2niix (Li et al., 2016) and denoised by MP-PCA (Veraart et al., 2016) in MRtrix3 (Tournier et al., 2019). We chose not to correct distortions related to echo-planar imaging (e.g., susceptibility and eddy-currents) because i) it remains a globally contentious issue (Veraart, 2022, Tax et al., 2022), ii) the most common software packages for this activity change diffusion parameter estimates from peripheral nerves (Wade et al., 2024) and, iii) such software are not available on clinical scanners.

### 2.5 Postprocessing

Data were imported to DSI Studio (Yeh, 2021). Diffusion was quantified using restricted diffusion imaging (Yeh et al., 2017) and reconstructed using Generalised Q-Sampling Imaging (Yeh et al., 2010), with a sampling length ratio of 1·25. We chose this model-free approach because peripheral nerves are more conspicuous on the resultant quantitative anisotropy (QA) maps than on traditional tensor-based maps (Fig. 2), it can also be applied to a variety of diffusion sampling schemes, the outputs are comparable to more complex q-space methods and it generates a spin-density function which is the closest to reality (Yeh et al., 2010).

**Fig. 1:**
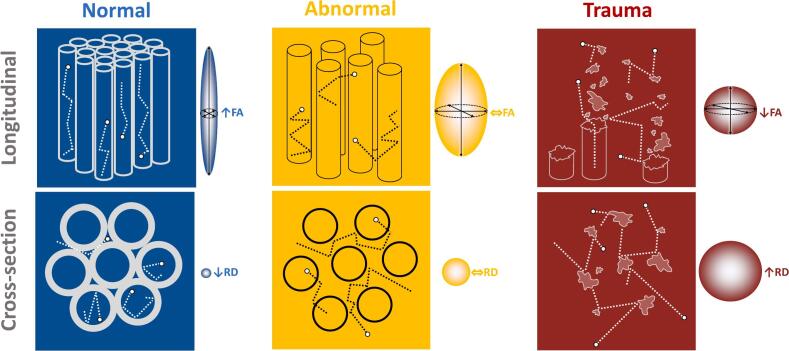
In blue is a schematic of a normal peripheral nerve (blue) which allows bidirectional diffusion of water in the long axis (axoplasmic flow) but restricts diffusion radially and thus, diffusion within nerves naturally has a high fractional anisotropy (FA) and low radial diffusivity (RD). In yellow is an example of an abnormal nerve, with deficiency in myelin and expanded endoneurium, which allows water to escape axons and diffuse more freely in the endoneurial space, leading to a reduction in anisotropy and rise in radial diffusivity. In red, an acutely injured nerve lacks microstructure meaning that water may diffuse more-or-less freely in all directions, rendering diffusion more isotropic. (For interpretation of the references to colour in this figure legend, the reader is referred to the web version of this article.)

**Fig. 2:**
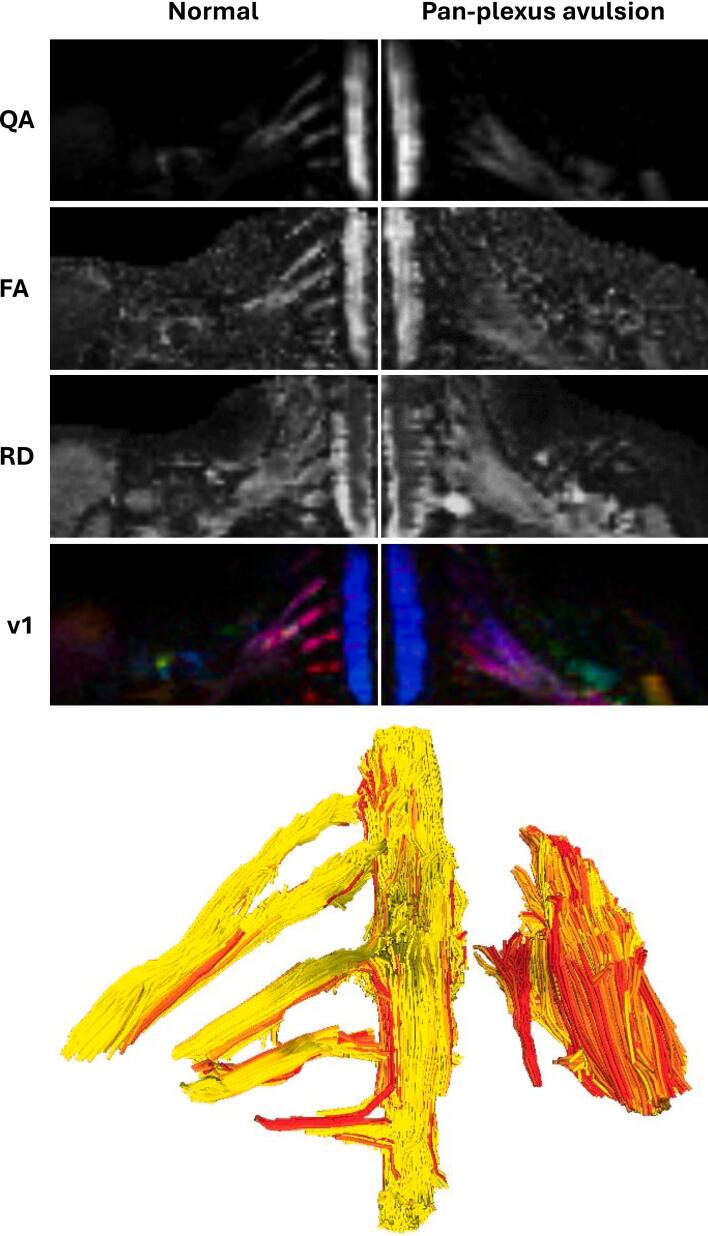
DTI acquired from a 44-year-old male with a left-sided pan-plexus palsy, 36 days after he was involved in a motorcycle accident. The QA, FA, RD and principal eigenvector (v1, with the colours red, green and blue representing diffusion in x, y and z directions, and the intensity scaled by quantitative anisotropy QA) maps are shown for the right and left sides. The tractogram is coloured from yellow (maximum FA 0.35) to red (maximum FA 0.25) to show that DTI detects the microstructural changes within the avulsed and retracted plexus. (For interpretation of the references to colour in this figure legend, the reader is referred to the web version of this article.)

### 2.6 Extraction of diffusion-related metrics

To extract metrics from the spinal nerve roots and mitigate partial volume effects, RoIs were manually drawn by RGW (9 years MRI experience) immediately distal to the intervertebral foramen; prior work has shown excellent interrater agreement for diffusion parameter extraction of spinal nerve roots (Sun et al., 2022, Wade et al., 2020, Chen et al., 2024) so multiple raters were not used. The RoI was limited to one voxel in one slice (2·5 × 2·5 × 2·5 mm, i.e., 6·25 mm^2^ of the cross sectional area) per root because the normal cross sectional area of the cervical spinal nerve roots C5-8 is 6 ∼ 10 mm^2^ (Fisse et al., 2021). The RoI was centred over the middle of the cross-section of the nerve, which typically also had the highest regional QA value (eFig. 1), to avoid partial volume effects. From each RoI we extracted these ‘RoI-based’ parameters: fractional anisotropy (FA), radial diffusivity (RD), axial diffusivity (AD) and mean diffusivity (MD).

### 2.7 Tractography

A deterministic fiber tracking algorithm (Yeh et al., 2013) was used with augmented tracking strategies (Yeh, 2020) to improve reproducibility. The anisotropy threshold was randomly selected between 0·5 and 0·7 Otsu threshold. The angular threshold was randomly selected from 45 to 90° (Wade et al., 2020). The step size was set to voxel spacing. The same RoIs used for RoI-based parameter extraction were used for tractography in “ROI” mode, albeit inflated to a volume of 7 voxels to capture tracts representing the full cross-sectional area of the roots (eFig. 2). Seeding continued until 250 tracts were calculated per root, or until 10 million seeds were surpassed without any streamlines generated. Tracks <30 mm were discarded. Topology informed pruning was applied with 2 iterations (Yeh et al., 2019). Duplicate tracts were discarded. The average diffusion parameters of the bundle of streamlines were then extracted, which we termed ‘bundle-based’ metrics (eFig. 3).

### 2.8 Reference standard

To determine the presence or absence of a root avulsions, surgical exploration of the supraclavicular brachial plexus was undertaken at the earliest opportunity (Wade et al., 2019). We defined avulsion as a binary state with implicit threshold. If the spinal foramina was empty (i.e. no identifiable nerve) then avulsion was diagnosed; equally, if there was a neural structure in the foramen but it was easily pulled away, then a concealed avulsion was diagnosed. In the case of exploration undertaken weeks-months after injury, avulsion was defined by a combination of: absent nerve roots in the foramina, attenuated and displaced scarred proximal nerve trunks or dorsal root ganglion; no identifiable nerve fascicles on exploration of the nerve root; empty proximal nerve sheaths. Intra-operative somatosensory evoked potentials (SEPs) were acquired from two patients and the findings are disclosed but they did not form part of the criteria for the diagnosis of avulsion. The C4 to T1 roots were explored in all participants.

### 2.9 Statistical analysis

Data were analysed using Stata v18/MP (StataCorp LLC, Texas). Scaled variables approximating the normal distribution are represented by the arithmetic mean (and standard deviation, SD) whilst skewed continuous data are represented by the geometric mean and 95 % confidence intervals (CI). Mean differences are given by Δ. The difference between sides (injured vs uninjured) are compared using paired t-tests. Inter-scan agreement (using RoI-based parameters) is summarised by Lin’s rho (using the *concord* function), which represents both precision and accuracy, whereby 1 is perfect concordance and 0 is no concordance. The level of statistical significance was set at 5 %. Data are shown as RainCloud (Allen et al., 2019) and Bland-Altman plots.

## 3 Results

14 males with tBPI (mean age 44 years, SD 14) had their first scan at a mean 18 days (CI 15–21) post-injury. After scanning, seven underwent exploration at a mean of 37 days (SD 20). Seven recovered spontaneously between the time of their scan and the planned surgery and defaulted to observation. Two patients had operatively confirmed root avulsions (one 45-year-old with pan-plexus avulsion and a 32-year-old with C5-7 avulsions).

### 3.1 RoI-based diffusion parameters

**Fig. 3:**
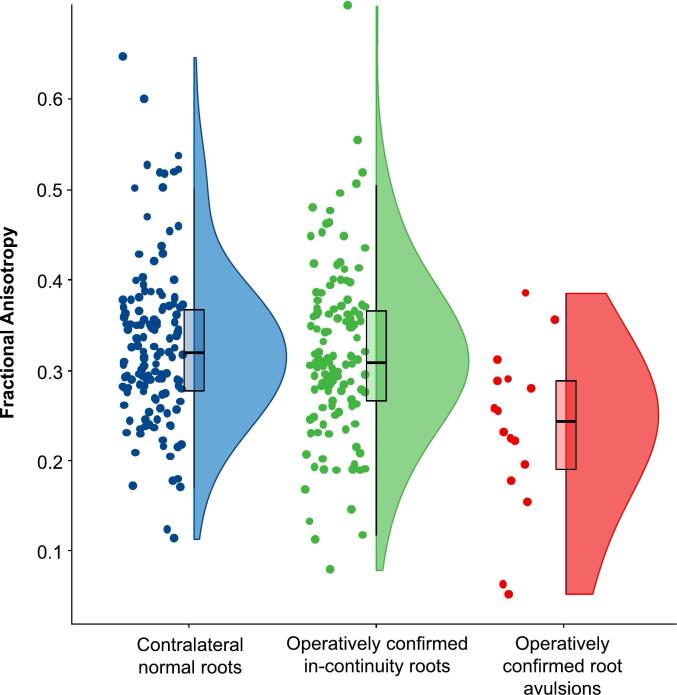
Diffusion within distal stump of avulsed roots was more isotropic than in-continuity injuries and contralateral normal roots.

At a mean of 18 days from injury (SD 17), diffusion was more isotropic in avulsed roots (Fig. 3, Table 1). Overall, root avulsions had 12 % lower FA than injured in-continuity roots (CI 5–19 %) and 14 % lower FA (CI 7–21 %) than the roots of the contralateral uninjured brachial plexus.

**Table 1:** RoI-based diffusion parameter estimates from the roots of the brachial plexus. Diffusivity values are in micrometres (x10^−3^ mm^2^/s*). Acronyms, depicted in Fig. 1: FA, fractional anisotropy; MD, mean diffusivity; RD, radial diffusivity; AD, axial diffusivity.

	Mean (SD)			
	FA	MD*	RD*	AD*
Operatively confirmed root avulsions	0·23 (0·09)	1·44 (0·35)	1·28 (0·37)	1·78 (0·36)
Operatively confirmed in-continuity roots	0·33 (0·09)	1·31 (0·27)	1·07 (0·26)	1·80 (0·33)
Contralateral healthy (uninjured) roots	0·32 (0·09)	1·32 (0·31)	1·09 (0·31)	1·78 (0·35)

**Fig. 4:**
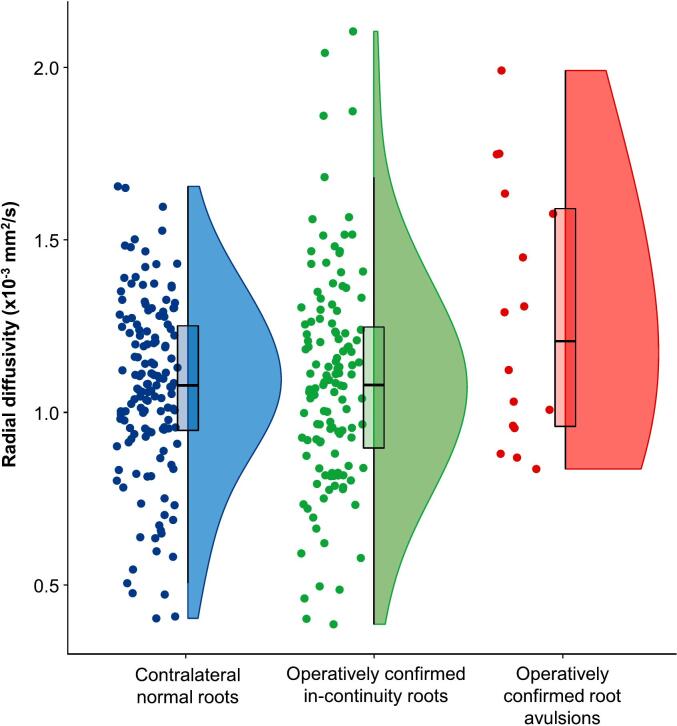
Diffusion perpendicular to the axis of the nerve (radially) was 27 % higher in avulsed roots than in-continuity injuries, and 34% higher than the contralateral normal roots.

Avulsed roots had higher radial diffusivity (Fig. 4, Table 1) than injured in-continuity roots (Δ 0·19 x 10^−3^ mm^2^/s [CI 0·003–0·48]) and contralateral uninjured roots (Δ 0·20 x 10^−3^ mm^2^/s [CI 0·02–0·40]). There were no differences in AD or MD between avulsed and in-continuity roots (Δ in AD −0·01 x10^−3^ mm^2^/s [CI −0·19, 0·17] and Δ in MD −0·13 x10^−3^ mm^2^/s [CI −0·30, 0·05]).

### 3.2 RoI-based diffusion parameters: Agreement of repeated scans

Before surgery, six patients were scanned twice at a mean interval of 21 days (SD 13). Overall, FA reduced by a mean of 0·024 (CI −0·003, 0·051) between scans (rho 0·312, p = 0·006). Importantly, these appeared to be due to changes within in-continuity roots (rho 0·118, Δ 0·025, p = 0·012) rather than avulsed roots. The FA of avulsed roots did not change between scanning sessions (rho 0·428, Δ 0·004, p = 0·202; eFig. 5) whilst we observed a relative five-fold reduction in FA within injured in-continuity roots (Δ 0·022, p = 0·330). Overall, concordance was lower for MD (rho 0·198, Δ 0·151), AD (rho 0·177, Δ 0·156) and RD (rho 0·225, Δ 0·149;) despite excellent agreement (eFig. 6).

### 3.3 Agreement between RoI-based and bundle-based parameter estimates

Streamlines could only be generated for 62 % of roots. In the instances that streamlines were generated, there was poor concordance (eFig. 7) between RoI-based and bundle-based estimates of FA (rho −0·019, Δ 0·175), MD (rho 0·023, Δ), AD (rho 0·215, Δ 0·236) and RD (rho 0·062, Δ 0·741).

### 3.4 SEPs

Two adults had SEPs measured intra-operatively. The first was a 51-year-old who sustained his injury after losing control of his motorcycle on a racetrack at 80mph. His clinical deficits were in the distributions of the upper trunk and posterior cord. The DTI parameters from his injured roots were within normal limits and not different to the uninjured side (injured roots mean FA 0.32 [SD 0.05] vs uninjured roots mean FA 0·33 [SD 0·07]; p = 0·559). At exploration, his roots were in-continuity but his SEPs were reduced in the C8 and T1 roots. By 13 months, he had made a full recovery. The second patient was a 30-year-old paedestrian who was struck by a car. He had an upper trunk deficit (absent elbow flexion and altered sensation in the C5/6 dermatomes). The DTI parameters from his roots were normal and not different to the uninjured side (injured roots mean FA 0.30 [SD 0.02] vs uninjured roots mean FA 0·33 [SD 0·02]; p = 0·209). His intraoperative SEPs were normal and exploration confirmed in-continuity roots. He later made a full recovery.

## 4 Discussion

DTI appears to be sensitive to the microstructural changes occurring in root avulsions within days-to-weeks of traumatic injury to the brachial plexus. Moreover, DTI appears to give equally accurate results whether performed early (e.g. within 3 weeks of injury) or later (e.g. 6 weeks after injury), so the timing of imaging can be tailored to the needs of the patient. Equally, serial scanning to detect changes in diffusion parameter estimates from the distal nerve stumps may help to differentiate ongoing degeneration from recovery. Overall, we suggest that DTI be routinely acquired alongside standard morphological sequences in clinical MRI protocols for tBPI.

The accuracy of MRI for tBPI root avulsion is modest (Wade et al., 2019), with a pooled sensitivity of 93 % and specificity of 72 %; however, since this meta work was published in 2018, several important advances have been made in the neurography. The use of intravenous gadolinium (Sneag et al., 2020, Han et al., 2022, Zhang et al., 2021) or Femuroxytol (Queler et al., 2021, Pedrick et al., 2023) to enhance short-tau inversion-recovery T2w fast spin echo neurography has been shown to improve the fidelity of the brachial plexus, principally by removing venous signal. Similar effects have been reported using combined spectral adiabatic inversion recovery fat-compression and improved motion-sensitized driven equilibrium pre-pulses (SHINKEI) to suppress fat, vascular and lymphatic signal around the brachial plexus (Zhang et al., 2024, Nair et al., 2021). The release of scanner-level deep-learning based reconstructions have substantially improved the signal-to-noise and so image quality of neurography, whilst also reducing the overall scan time (Sneag et al., 2023, Hu et al., 2023, Pribowo et al., 2023). Overall, these developments have improved the clarity of magnitude images and enabled the visualisation of smaller terminal branches of the brachial plexus, as well as better visualisation of the proximal elements which would otherwise have been obscured by signal from non-neurological tissues. Notwithstanding, all these enhancements and developments apply to T2w morphological imaging which still only generates greyscale images. Such images require a clinician to make a subjective assessment and reach a diagnosis with implicit threshold. The inherent weakness of traditional morphological imaging is the fundamental lack of objective quantitative data. Therefore, we suggest that protocols be complemented by quantitative MRI sequences designed to acquire objective metrics from nerves themselves (e.g. via diffusion-weighted MRI (Breckwoldt et al., 2015) and their end organs (e.g., muscle (Tan et al., 2022, Tan et al., 2022, Tan et al., 2022, Campbell et al., 2024). We believe that the synergy of morphological imaging with quantitative metrics, all derived from MRI, represents the next frontier in the assessment of neuropathy.

Animal studies have shown that diffusion becomes more isotropic, with a rise in RD and fall in FA within the distal stump of injured peripheral nerves in rats (Farinas et al., 2020, Andersson et al., Manzanera Esteve et al., 2021, Farinas et al., 2020, Manzanera Esteve et al., 2019, Afshari et al., 2018, Wu et al., 2017) and rabbits (Wan et al., 2021, Farinas et al., 2019). These changes are proportional to the severity of nerve injury (Manzanera Esteve et al., 2021, Farinas et al., 2020) and associated with limb function (Farinas et al., 2020, Wu et al., 2017). Moreover, diffusion-weighted MRI is equally sensitive to axonal regrowth (Afshari et al., 2018, Farinas et al., 2019) across the zone of injury and distally (Voser et al., 2024). To-date, there is limited data on DTI in human peripheral nerve injury. Pridmore et al (2020) showed that 3 patients with traumatically injured peripheral nerves exhibited lower FA and higher diffusivity (Pridmore et al., 2021) and Voser et al. (2024) showed more isotropic diffusion at the site of median nerve division and distally, which regressed to near-normal 12 months after repair (Voser et al., 2024). Our findings are commensurate with the wider literature on DTI in traumatic nerve injury in both animals and humans. Further work is required to understand the relationship between diffusion signals and peripheral nerve injuries in humans.

Overall, we found tractography to be unhelpful in the assessment of the tBPI. This is because i) in one third of patients the tracking algorithm, despite being the most reliable deterministic method globally and widely-used in brain imaging, failed to generate streamlines representing the roots and so metric extraction was impossible, ii) tracking was computationally intensive and time consuming, extraction of RoI-based metrics took a few minutes per patient whereas generating and pruning streamlines sometimes took hours, iii) bundle-based metrics had poor agreement with RoI-based metrics and, iv) streamlines are not nerves and equally, the presence of a streamline does not mean that the nerve is in-continuity or healthy. At best, streamlines may be coloured to show the anatomical location of microstructural abnormalities inferred by isotropy. At worse, false-positive streamlines could lead clinicians to believe nerves are normal when they are not. Overall, we feel that tractography should not be used in the diagnosis of tBPI until more work is done to understand how it should be performed and interpreted. Importantly, the international community still cannot reach consensus on the ideal acquisition parameters, preprocessing steps and tractography methods for the brain. Therefore, we suggest that clinicians and researchers wishing to acquire DTI from peripheral nerves limit its used to maps (QA, FA, RD, etc) for now and consider the below recommendations for sequence optimisation. As tensors are robust to varying b-values (in the hindered range) we suggest a b-value of 300–800 mm^2^/s; smaller b-values enable a shorter TE which improves SNR and mitigates T2 shine-through (at the expense of less diffusion-weighting), and may enable vendor-specific options to improve image quality and reduce distortions. Given the tortuous anatomical course and microstructural complexity (fascicular sharing) of the roots, we suggest at least 30 non-colinear directions are used, which should also boost SNR, with b0s interleaved – this enables corrections for motion with a negligible time cost. Given that data distal to the roots is difficult to resolve anatomically, we recommend experimentation with reduced field-of-view products (e.g. Siemen’s ZOOMit, GE’s FOCUS or Philip’s iZOOM) which may reduce the echo-train-length and thus, shorten the TE to improve distortions and SNR. The number of averages should be set to achieve adequate SNR and if two or more are needed, then readers may consider reversing the phase-encoding polarity to later correct for distortions related to susceptibility and eddy-currects (Wade et al., 2024).

### 4.1 Limitations

Longitudinal research on adults with tBPI is already difficult for many reasons; the addition of the Covid-19 pandemic compounded difficulties, adversely affecting recruitment and retention. NIHR-funded research was paused temporarily, referrals from regional centres reduced and patients were reluctant to travel for scans. Nonetheless, we recruited a representative sample of adults to demonstrate the potential utility of DTI. More data on this topic is needed to reach reliable conclusions.

We chose to denoise the magnitude images using MP-PCA (Veraart et al., 2016) in MRtrix3 (Tournier et al., 2019) because this is the best performing algorithm to-date (Sneag et al., 2020); however, other denoising methods are available and the choice is likely to affect parameter estimates. The effect of in-line vendor denoising packages on diffusion parameter estimates remains unknown. Equally, newer acceleration technologies (e.g. simultaneous multislice) may introduce bias .

We elected not to perform distortion correction (e.g., using FSL's TOPUP and eddy) for the reasons described in the methods, which may be controversial, and may impact the results and generalisability of our findings. We used a custom diffusion waveform and vector scheme to optimise distortions, artefacts and postprocessing; the performance of default vendor schemes or otherwise may be different. Fascicular exchange occurs throughout the brachial plexus and so it is plausible that >1 fibre orientation exists within a given voxel; this means that the treatment of diffusion as Gaussian in DTI may be inadequate and future work should consider the use of non-Gaussian methods for greater sensitivity to restricted diffusion. We appreciate that our data may be confounded by pulsatile CSF and blood flow, respiration and patient movement, all of which can be ameliorated during acquisition or corrected for after-the-fact, albeit the effects of this on the true values are unknown.

## 5 Conclusions

We suggest that clinicians add diffusion-weighted imaging, at least DTI, to their clinical protocols for the assessment of adults with traumatic brachial plexus injuries. This additional information may improve the overall diagnostic accuracy of MRI for detecting root avulsion, meaning that patients who need early reconstruction might be better identified and treated sooner.

## Ethical approval

Approval was gained from the National Research and Ethics Service of the United Kingdom (IRAS project ID 260445, HRA REC reference 19/NW/0324).

## CRediT authorship contribution statement

**Ryckie G. Wade:** Conceptualization, Data curation, Formal analysis, Funding acquisition, Investigation, Methodology, Project administration, Resources, Software, Supervision, Validation, Visualization, Writing – original draft, Writing – review & editing. **Irvin Teh:** Writing – review & editing, Validation, Supervision, Software, Resources, Project administration, Methodology, Investigation, Formal analysis, Data curation. **David Shelley:** Writing – review & editing, Supervision, Resources, Project administration, Data curation. **Robert D. Bains:** Writing – review & editing, Supervision, Project administration. **James D. Bedford:** Writing – review & editing, Project administration, Data curation. **Lucy E. Homer Newton:** Writing – review & editing, Project administration, Data curation. **Chye Yew Ng:** Writing – review & editing, Project administration, Data curation. **Grainne Bourke:** Writing – review & editing, Visualization, Validation, Supervision, Resources, Project administration, Methodology, Investigation, Funding acquisition, Data curation, Conceptualization.

## Funding

Ryckie Wade captured this data during a Doctoral Research Fellowship funded by the National Institute for Health Research (NIHR, DRF-2018-11-ST2-028) and analysed it during an Academic Clinical Lecturer funded by the NIHR (CL-2021-02-002). This research is also supported by the NIHR Leeds Biomedical Research Centre and University of Leeds Advanced Imaging Centre, which is funded by the Medical Research Council (MR/M008991/1) with support from the British Heart Foundation (BHF-SP/14/7/31351) and Arthritis Research UK (ARUK-21078). The views expressed are those of the author(s) and not necessarily those of the United Kingdom’s National Health Service, NIHR or Department of Health.

## Declaration of competing interest

The authors declare that they have no known competing financial interests or personal relationships that could have appeared to influence the work reported in this paper.

## Data Availability

Data will be made available on request.
