# Supplementary material for: Diffusion tensor imaging at 3T for diagnosing root avulsion in adults with acute traumatic brachial plexus injuries
Source: Neuroimage Clin. 2025 May 21;47:103806. doi: 10.1016/j.nicl.2025.103806 (PMC12166390; doi:10.1016/j.nicl.2025.103806)
Supplement: Supplementary Data 1 [file mmc1.docx]

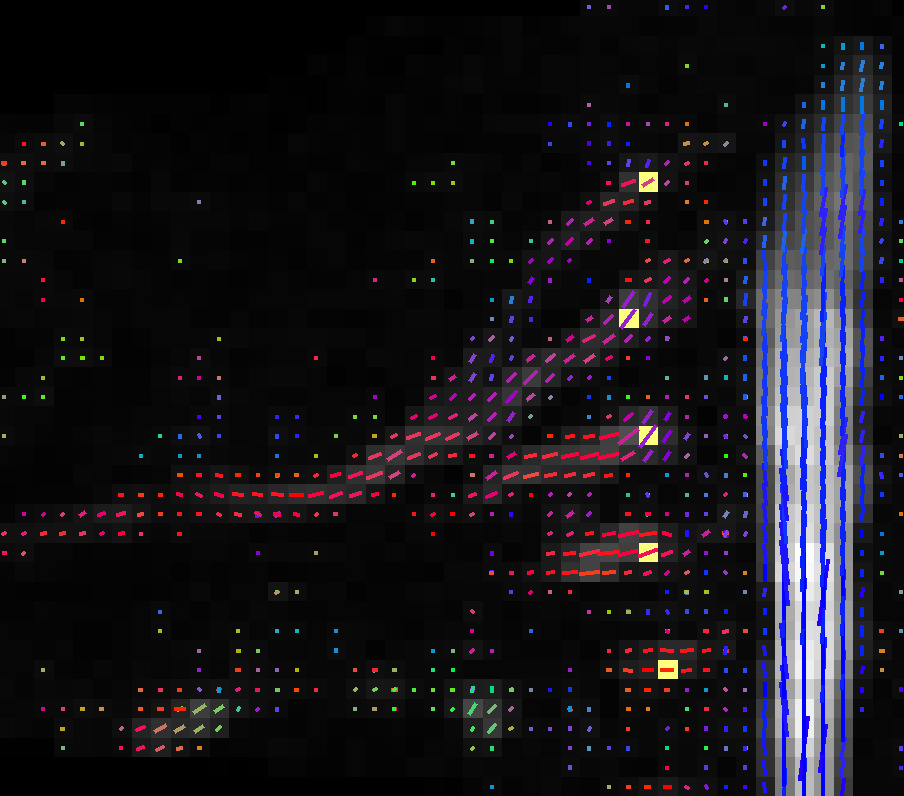

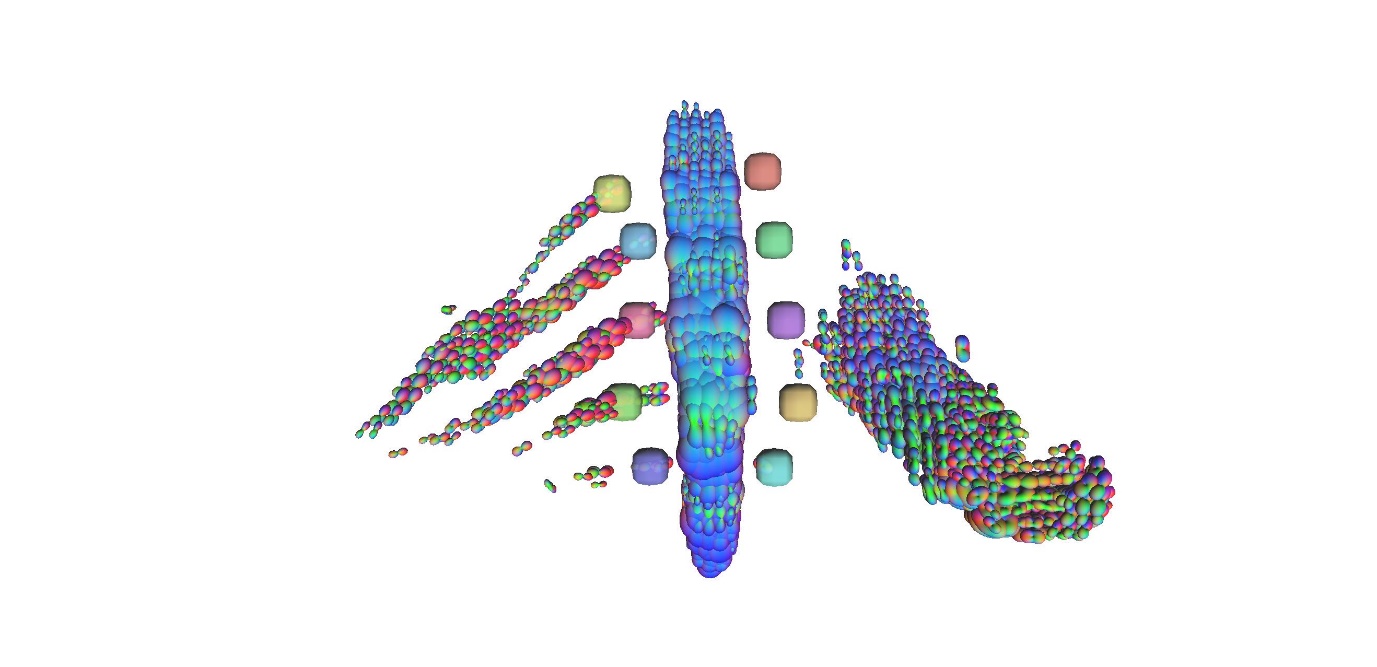


**eFigure 1**. RoI placements. The panel on the left shows the stick-glyphs of GQI reconstruction of a right-sided (normal) brachial plexus and how the RoIs measing a single voxel were positioned centrally on each root. The panel on the left shows the orientation-distribution functions (ODF) glyphs of a patient with left-sided panplexus avulsion which has retracted into the supraclavicular fossa; in this case, the RoIs are position symmetrically with respect to the normal contralateral side given the absence of anisotropic voxels and ODFs to guide placement. Glyphs are coloured according to the principal eigenvector (v1), with red, green and blue representing diffusion in x, y and z directions.


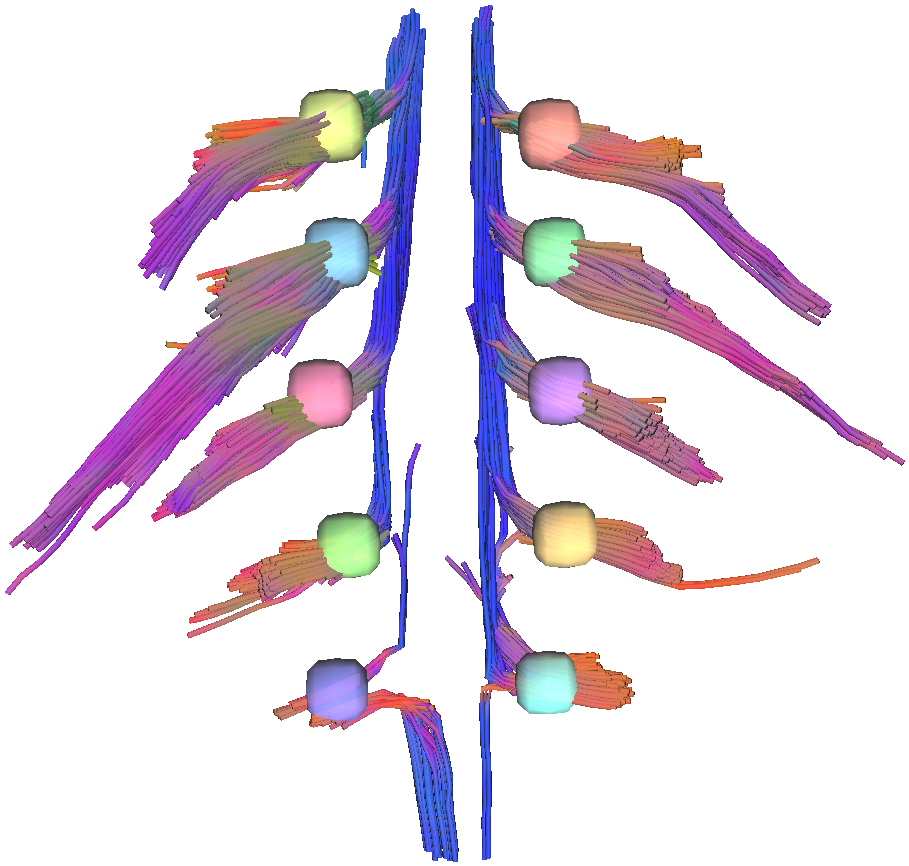


**eFigure 2.** A tractogram showing the placement of RoIs used to generate streamlines, which are coloured according to the principal eigenvector (v1), with red, green and blue representing diffusion in x, y and z directions.


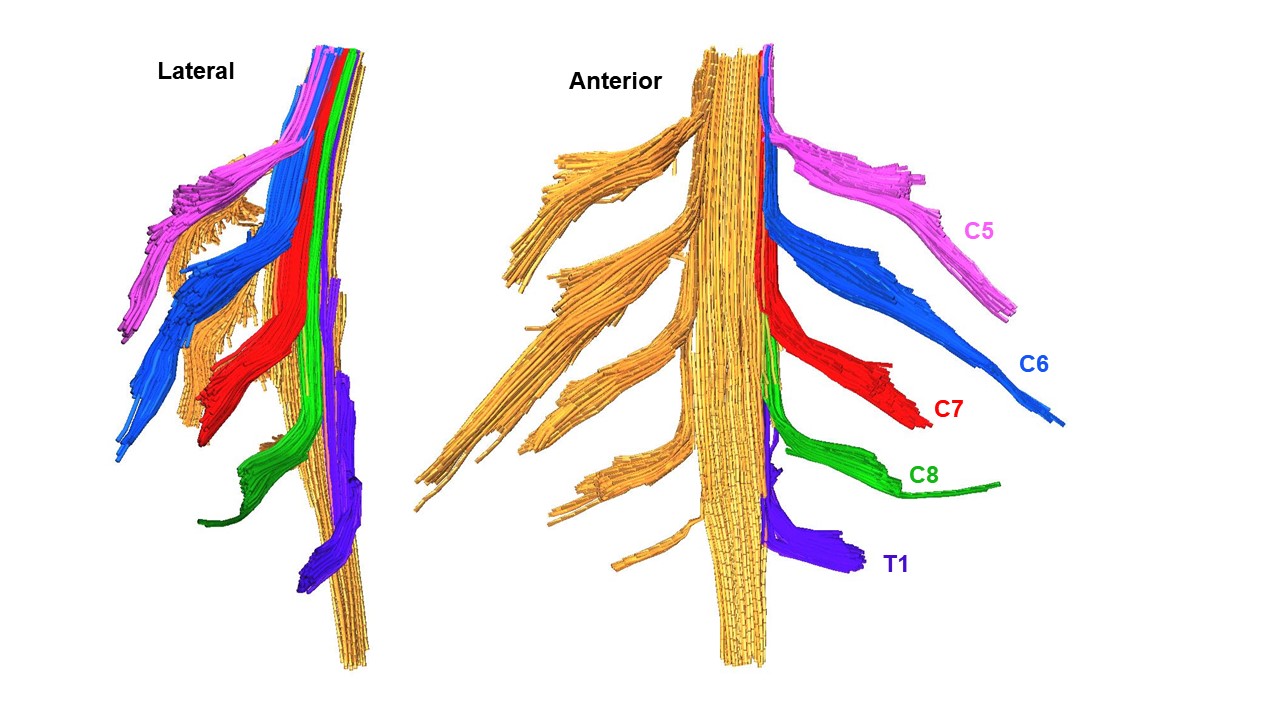


**eFigure 3**. A tractrogram (seen from the left-lateral and anterior views) with the bundles of streamlines coloured to show the root levels. Bundle-based metrics were extracted from each bundle. Note, streamlines representing the spinal cord are shown here for context only.


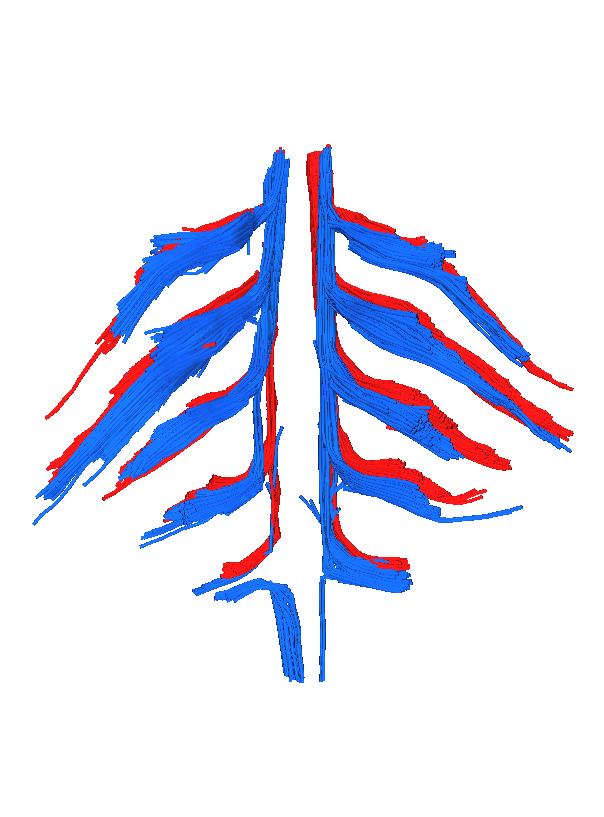


**eFigure 4**. Tractography from a 50-year-old cricket coach 7-days (red) and 18-days (blue) following a left reverse total-shoulder arthroplasty which caused a sided pan-plexus palsy. He started to recover before his planned date for exploration and so surgery was cancelled. Ultimately, he made a full recovery.


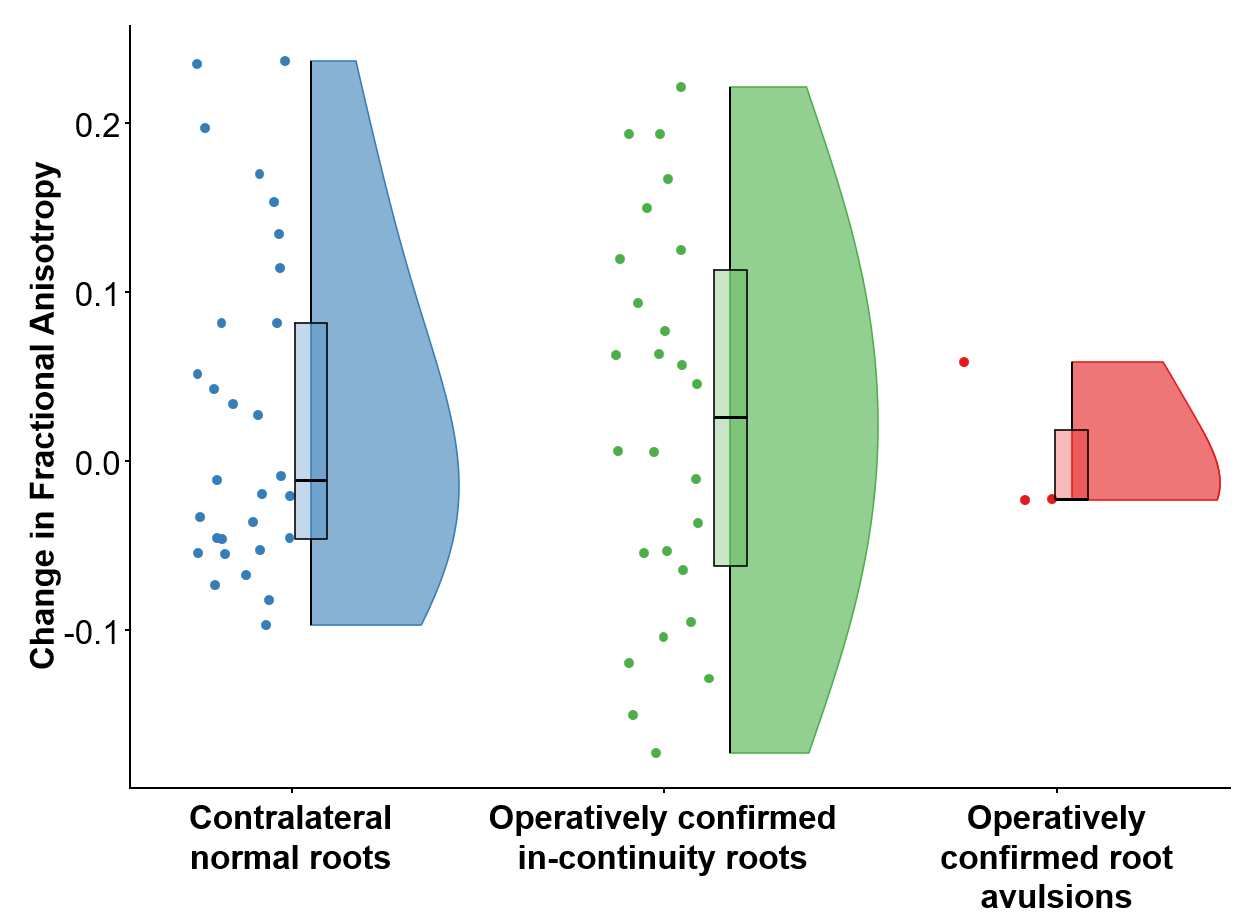


**eFigure 5**. A RainCloud plot showing no significant change in FA between repeated scanning sessions in avulsed or in-continuity spinal nerve roots. Overall, the mean change between scans was 2% (CI -3, 5).

**eFigure 6.** Bland-Altman plots showing the inter-sessional agreement for 6 patients who were scanned twice at a mean interval of 21 days (SD 13). RoI-based estimates of FA, and MD (x10^-3^ mm^2^/s), AD (x10^-3^ mm/s^2^) and RD (x10^-3^ mm/s^2^).

**eFigure 7.** Bland-Altman plots showing poor agreement between RoI-based estimates and bundle-based estimates of FA, and MD (x10^-3^ mm/s^2^), AD (x10^-3^ mm/s^2^) and RD (x10^-3^ mm^2^/s). These are data from 6 patients who were scanned twice at a mean interval of 21 days (SD 13).
